# Amino Acid Isotope Incorporation and Enrichment Factors in Pacific Bluefin Tuna, *Thunnus orientalis*

**DOI:** 10.1371/journal.pone.0085818

**Published:** 2014-01-22

**Authors:** Christina J. Bradley, Daniel J. Madigan, Barbara A. Block, Brian N. Popp

**Affiliations:** 1 Department of Oceanography, University of Hawaii, Honolulu, Hawaii, United States of America; 2 Tuna Research and Conservation Center, Pacific Grove, California, United States of America; 3 Stanford University, Hopkins Marine Station, Pacific Grove, California, United States of America; 4 Monterey Bay Aquarium, Monterey, California, United States of America; 5 Department of Geology and Geophysics, University of Hawaii, Honolulu, Hawaii, United States of America; Technical University of Denmark, Denmark

## Abstract

Compound specific isotopic analysis (CSIA) of amino acids has received increasing attention in ecological studies in recent years due to its ability to evaluate trophic positions and elucidate baseline nutrient sources. However, the incorporation rates of individual amino acids into protein and specific trophic discrimination factors (TDFs) are largely unknown, limiting the application of CSIA to trophic studies. We determined nitrogen turnover rates of individual amino acids from a long-term (up to 1054 days) laboratory experiment using captive Pacific bluefin tuna, *Thunnus orientalis* (PBFT), a large endothermic pelagic fish fed a controlled diet. Small PBFT (white muscle δ^15^N∼11.5‰) were collected in San Diego, CA and transported to the Tuna Research and Conservation Center (TRCC) where they were fed a controlled diet with high δ^15^N values relative to PBFT white muscle (diet δ^15^N∼13.9‰). Half-lives of trophic and source amino acids ranged from 28.6 to 305.4 days and 67.5 to 136.2 days, respectively. The TDF for the weighted mean values of amino acids was 3.0 ‰, ranging from 2.2 to 15.8 ‰ for individual combinations of 6 trophic and 5 source amino acids. Changes in the δ^15^N values of amino acids across trophic levels are the underlying drivers of the trophic ^15^N enrichment. Nearly all amino acid δ^15^N values in this experiment changed exponentially and could be described by a single compartment model. Significant differences in the rate of ^15^N incorporation were found for source and trophic amino acids both within and between these groups. Varying half-lives of individual amino acids can be applied to migratory organisms as isotopic clocks, determining the length of time an individual has spent in a new environment. These results greatly enhance the ability to interpret compound specific isotope analyses in trophic studies.

## Introduction

There are a number of novel tools available to trace nutrient movement through food webs or ecosystems. Stable isotope analysis has gained favor for its ability to detect the integration of dietary information over space and time [1]–[3]. Amino acid compound specific isotopic analysis (AA-CSIA) has shown distinct potential in elucidating information about both nutrient sources and trophic interactions from a single organism [4]–[9]. Calculations of trophic position from isotopic analysis of bulk tissue require knowledge of baseline isotopic values to correct for spatial and temporal variations in the values of compounds such as NO_3_, NO_2_, NH_4_ and total dissolved inorganic carbon, which are propagated up food webs. The approach of CSIA allows information about ecosystem interactions to be gathered from discrete samples of targeted species without the need for exhaustive surveys typically associated with the use of bulk isotopic and gut content analyses.

Through the application of CSIA, one can reveal and compare both the sources of nutrients and how those nutrients are utilized within each system. CSIA uses the ratio of ^15^N/^14^N (*δ* ^15^N) and ^13^C/^12^C (*δ* ^13^C) in specific compounds within tissues to reveal information about trophic and ecological relationships. Nitrogen isotopic values of amino acids obtained from CSIA have revealed several amino acids that retain the same δ^15^N values across multiple trophic steps [4], [5], termed “source” amino acids. “Trophic” amino acids fractionate ^15^N consistently with each trophic step due to *de novo* synthesis or transamination and deamination during assimilation, yielding ^15^N contents enriched compared to source amino acids [4], [5]. Evaluating a range of organisms from photoautotrophs to secondary consumers, Chikaraishi et al. [7] confirmed the enrichment in ^15^N from food source to consumer in trophic amino acids and little change in source amino acids [4], [6], [7], [10], [11]. The AA-CSIA method allows not only the calculation of trophic position, but also comparisons of source δ^15^N values between similar organisms to evaluate food web and migratory dynamics [5]–[8].

The rate of incorporation of a new isotopic composition into the tissues of an organism determines how long an immigrant to a region will be distinguishable from a long-term resident [12]. Isotopic incorporation is thought to relate to tissue turnover; tissues with higher protein turnover rates tend to incorporate changes in diet more quickly than slower growing tissues [13]–[17]. Following a change in diet, two processes dominate the shifts of the isotopic compositions of tissues: (1) metabolic turnover through the breakdown of older tissues and replacement with newly synthesized ones and (2) growth of new tissue post diet shift [18]–[20].

As the magnitude of isotopic enrichment in amino acids has been shown to be governed by biochemical processes at the tissue level [7], it is likely that rates of isotopic turnover in amino acids are similarly controlled. Controlled diet experiments that track shifts in nitrogen isotopic values during uptake or consumption and subsequent assimilation can be used to measure turnover rates and trophic discrimination factors (TDFs). These values are critical to accurately interpreting stable isotope data in ecological studies. While it remains important to look at bulk tissue isotopic values to determine the roles of the rates of growth and metabolism, it is not known how, if at all, these impact the turnover at the level of specific amino acids. Therefore, in order to model trophic interactions using stable isotope analysis of specific compounds, it is crucial to better understand the incorporation rates of specific amino acids into tissues, coupling this with bulk tissue information and tissue biochemistry.

Pacific bluefin tuna, *Thunnus orientalis*, (PBFT) used in this study were obtained from the Tuna Research and Conservation Center (TRCC) and Monterey Bay Aquarium (MBA), which had been held in captivity from 11 to 1054 days [21]. This provided the opportunity for long-term tracking of changes in isotopic composition of amino acids in muscle tissue following an isotopic change in diet, from a wild to a controlled diet. The dataset is the longest timescale available for a large pelagic fish fed a controlled diet. The goals of this study were to calculate turnover rates of individual amino acids in white muscle tissue of PBFT, determining the relative contributions of growth and metabolism to turnover. Additionally, TDFs were calculated for amino acids from individuals that had reached steady state with the isotopic composition of the controlled diet. The use of individual amino acid turnover rates to develop an isotopic clock, along with TDF values, can be used to more precisely study the migration and ecology of PBFT.

## Methods

### Captive Husbandry of Tunas

Juvenile Pacific bluefin tuna, *Thunnus orientalis*, were collected by hook and line off the coast of San Diego, CA, and held in tanks at the Tuna Research and Conservation Center (TRCC) in Pacific Grove, CA. Additionally, a number of bluefin tuna, ranging in age from 1 – 2 years, were caught by hook and line and samples of dorsal muscle tissue were immediately collected. Curved fork length (CFL) was measured at the time of collection and, for captive reared fish, at the time of death, which was either by natural mortality or having been sacrificed for experiments. A detailed description of handling and rearing methods can be found in Madigan et al [21]. Tunas in captivity were fed a consistent mixture of sardine, *Sardinops sagax*, squid, *Doryteuthis opalescens*, and gelatin (by mass: 70%, 21%, and 9%, respectively) with bulk weighted mean δ^15^N values (13.88±0.65 ‰) higher than white muscle (WM) tissues (11.80±0.24 ‰) of juvenile year class one and two (YC1 and YC2) PBFT captured off southern California.

Captive and wild PBFT were sampled for bulk WM tissue and results can be found in Madigan et al (2012). From the entire dataset (1-2914 days), AA-CSIA were measured on a subset of these samples representing 13 individual time points (individual fish) ranging in time in captivity from 11–1054 days. Additionally, AA-CSIA were measured in 8 wild PBFT ranging in age from 489 to 876 days [21]. Wild PBFT were aligned with captive fish by estimating their residency time in the California Current Large Marine Ecosystem (CCLME) using fish size [22], where the smallest sampled individual (61.6 cm) represented an approximate starting value for t (t = 0 days).

### Amino Acid Isotope analysis

White muscle (WM) tissue was collected from the hypaxial musculature of PBFT under the first dorsal fin and ∼10 cm below the skin. Of the captive feed, a section of dorsal WM was taken from sardines, and a section of mantle was taken from squid, removing the outer membrane (n = 2 for each feed item). Tissues were frozen at −80°C and subsequently lyophilized and ground to a homogenous powder for isotope analysis.

Muscle tissue samples were hydrolyzed and amino acids were derivatized prior to nitrogen isotope analysis using established methods [5], [8]. Dried, ground tissue was hydrolyzed (6N HCl) at 150°C for 70 minutes [23]. Acid hydrolysis under these conditions converts glutamate and aspartate to glutamic and aspartic acid, and destroy tryptophan and cystine. Samples were redissolved in 0.01N HCl, and purified by passage through a 0.45 µm hydrophilic filter and amino acids were further purified using cation-exchange chromatography (Dowex 50WX8-400) following the recommendation of Metges et al [24]. Amino acids were esterified by heating at 110°C for 60 minutes in 4∶1 isopropanol: acetyl chloride. Following esterification, amino acids were acylated by heating samples at 100°C for 15 minutes in 3∶1 methylene chloride:trifluoracetic anhydride. Samples were further purified by solvent extraction [25] using 2∶1 P-buffer: chloroform mix (P-buffer: KH_2_PO_4_+Na_2_HPO_4_ in Milli-Q water, pH 7). A final acylation step was repeated to complete the derivatization process. Samples were stored at −20°C in 3∶1 methylene chloride:TFAA for up to 6 months before isotope analysis.

The δ^15^N values of derivatized samples were determined using a Delta V mass spectrometer interfaced to a Trace GC gas chromatograph through a GC-C III combustion furnace (980°C), reduction furnace (650°C), and liquid nitrogen cold trap via a GC-C III interface. Samples were injected (split/splitless injector in splitless mode) onto a *forte* BPx5 capillary column (60 m×0.32 mm×1.0 µm film thickness) at an injector temperature of 180°C with a constant helium flow rate of 1.4 ml min^–1^. The column was initially held at 50°C for 2 min and then increased to 190°C at a rate of 8°C min^–1^. Once at 190°C, the temperature was increased at a rate of 10°C min^–1^ to 300°C where it was held for 7.5 min. All samples were analyzed at least in triplicate and measured δ^15^N values were normalized to the known nitrogen isotopic composition of internal references norleucine and aminoadipic acid co-injected with each sample. The standard deviation of δ^15^N values derived from multiple analyses averaged 0.52‰ and ranged from 0.03‰ to 1.2‰. Isotopic compositions of sixteen amino acids were obtained using this method. Nitrogen isotope ratios are described by:
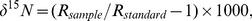
(1)where *R_sample_* is the ratio ^15^N∶^14^N within the sample, and *R_standard_* is the isotope standard Air. Isotope values are reported as permil (‰).

### Turnover estimation

We used two models to estimate amino acid turnover and calculate the half-life of each amino acid in muscle tissue. An exponential fit model as used previously [21], [26]–[29] is given by the equation:
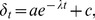
(2)where δ_t_ is the isotope value of the amino acid changing with time *t* and λ is a data-derived first-order rate constant. Parameters *a* and *c* represent the difference between initial and final steady-state δ^15^N values and the data-derived final steady-state δ^15^N value, respectively [27].

Additionally, we modeled amino acid turnover using the reaction progress variable as described in [13]:
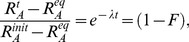
(3)where the nitrogen isotopic composition of a specific amino acid is *R_A_^t^* at any given time, *t*, *R_A_^eq^* at the data-derived equilibrium, or *R_A_^init^* at the initiation of the experiment. F = 0 at the beginning of the experiment and F approaches 1 as the animal reaches an isotopic steady state with the new diet (Cerling et al. 2007). The use of multiple tissue compartments to model incorporation of isotopes into tissue often more accurately describes the dynamics of turnover than the first-order kinetics of a single compartment model [30]. The reaction progress variable is used here to evaluate the appropriateness of either a single or multi-compartment model to describe the nitrogen isotopic turnover of individual amino acids in PBFT white muscle tissue. A single compartment model for isotopic turnover is described by a linear fit of ln(1-F) versus time, where the slope is equal to -λ.

The amino acid isotope-specific half-life (*t*_0.5_) is then calculated:
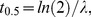
(4)for different *λ* values derived for δ^15^N value of each amino acid. For a given percentage (*α*) of complete turnover, the time needed was calculated using a modified equation from [20]:
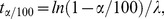
(5)where *t*_α/100_ is the time needed to attain *α*% turnover and *λ* is the data-derived first-order rate constant.

We compared the turnover rates of trophic and source groupings of amino acids to derive the best combination for use in interpreting trophic ecology of PBFT. Discussion of turnover results focus on the first approximately three years of an eight year study, utilizing only the time period during which measured growth of PBFT was linear. The change in the rate of tuna growth after three years corresponded to movement of the tuna to larger tanks at the Monterey Bay Aquarium and a possibly less controlled diet, which lead to an increase in tuna growth rate. Nevertheless, this limited time selection was sufficient to achieve 95% turnover of 8 of the 9 amino acids; threonine reached steady state ∼30 d after the final selected time point.

### Trophic Discrimination and Enrichment Factors (TDF and TEF)

TDF values (Δ_AA_) are calculated for each amino acid according to the equation:
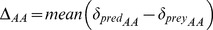
(6)where Δ*_AA_* represents the amino acid-specific TDF, *δ_predAA_* is the nitrogen isotope value of a specific amino acid for each individual tuna at steady-state with diet, defined by the asymptotic values in the model, and *δ_preyAA_* is a weighted mean amino acid nitrogen isotope value, by proportional mass of each food item (squid, sardine, and gelatin) of the control diet. Steady-state was determined from the time after which an individual amino acid achieved 95% turnover (t_0.95_), as defined by the model. Food items were sampled periodically throughout 2008–2010 to ensure consistency of food isotopic compositions. Error for *δ_preyAA_* was propagated using the uncertainty in each trophic or source amino acid between samplings, as well as the measured reproducibility of sample injections. Error for *δ_predAA_* was defined by the confidence intervals of the steady state δ^15^N values derived from the modeled isotopic incorporation (eqn. 2).

Trophic enrichment factors (TEFs) were calculated from the equation:
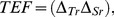
(7)where Δ*_Tr_* and Δ*_Sr_* are the TDFs of a single or weighted mean trophic amino acid and source amino acid, respectively. Combinations of five trophic (Ala, Glu, Leu, Pro, Val) and six source amino acids (Gly, Lys, Phe, Ser, Thr, Tyr) were used for calculations as well as the weighted mean of each group.

We compared our experimentally-derived TEFs to those found in Chikaraishi et al. [7] for application in estimating trophic position (TP) from the equation:
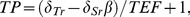
(8)where δ*_Tr_* and δ*_Sr_* are the nitrogen isotopic compositions of one or a mean of trophic and source amino acids, respectively, and *β* is the difference between trophic and source amino acids in primary producers (Chikaraishi et al. 2009). Appropriate values of β for each combination of trophic and source amino acids were calculated from results in Chikaraishi et al. [6], [7], [31].

### Effects of growth

From Hesslein et al. [32] we can describe the first-order growth rate constant, λ, as:
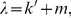
(9)where *k′* is the growth rate constant derived from the relationship of relative growth (W_R_), or the relative gain in mass, with time in captivity, as described in Madigan et al. [21]. W_R_ was calculated as the ratio of final to initial mass of the experimental organism [21].

Applying eq. 2 to the change in isotopic composition of individual amino acids with W_R_, we calculate the relative growth needed to achieve α percent turnover (Buchheister and Latour 2010):
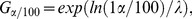
(10)where *λ* is the data-derived rate constant.

### Ethics Statement

Permits for collection of animals were provided by California Fish and Game. All procedures used in these experiments were approved by the Administrative Panel on Laboratory Animal Care (APLAC) of Stanford University.

## Results

Of the sixteen amino acids which are retrievable using the CSIA method outlined above, twelve were measured reliably in all samples (Table 1). Those twelve are alanine (Ala), glycine (Gly), threonine (Thr), serine (Ser), valine (Val), leucine (Leu), aspartic acid (Asp), proline (Pro), glutamic acid (Glu), phenylalanine (Phe), lysine (Lys), and tyrosine (Tyr). Histidine (His) was measured in all but one sample, and isoleucine (Ile), methionine (Met), and arginine (Arg) were measured in fewer than 50% of samples.

**Table 1:** δ^15^N values of amino acids and time in captivity for Pacific bluefin tuna (*Thunnus orientalis*).

			Trophic Amino Acids								Source Amino Acids					
Time in captivity (d)	CFL initial (cm)	CFL death (cm)	Ala (SD)	Asp (SD)	Glu (SD)		Leu (SD)†	Pro (SD)		Val (SD)†	Gly (SD)	Lys (SD)†	Phe (SD)	Ser (SD)	Thr (SD)†	Tyr (SD)
11	67.0	67.5	24.2 (0.8)	23.2 (0.4)	24.0 (0.5)	21.2 (0.8)			16.8 (0.9)	19.3 (0.6)	−3.2 (0.9)	3.9 (0.4)	8.4 (0.2)	0.4 (0.5)	−21.9 (0.5)	11.2 (0.5)
52	66.5	71.0	27.6 (0.2)	26.6 (0.6)	26.9 (0.4)	25.6 (0.4)			23.3 (0.8)	21.9 (0.3)	−2.4 (0.4)	6.0 (0.4)	7.0 (0.7)	3.0 (0.5)	−23.8 (0.9)	9.3 (1.0)
58	67.0	71.0	28.1 (0.5)	26.8 (0.2)	25.6 (0.3)	25.1 (0.7)			23.4 (0.1)	24.9 (0.9)	0.3 (0.3)	5.9 (0.8)	7.4 (0.4)	6.3 (0.7)	−21.1 (0.7)	14.7 (0.1)
95	75.0	82.0	25.9 (0.7)	22.8 (0.4)	26.0 (0.6)	25.1 (0.7)			20.6 (0.2)	24.7 (0.7)	−3.3 (0.1)	5.0 (0.5)	9.9 (0.3)	3.5 (0.9)	−22.4 (0.6)	14.3 (0.5)
141	70.0	73.3	28.8 (0.3)	27.3 (0.2)	26.2 (0.2)	25.3 (0.1)			25.3 (0.4)	22.6 (0.4)	0.6 (0.4)	6.5 (0.6)	7.2 (0.7)	5.4 (0.5)	−22.8 (0.7)	10.9 (0.3)
219	67.0	75.4	29.8 (0.4)	27.4 (0.3)	27.1 (0.3)	26.7 (0.3)			24.6 (0.2)	26.3 (0.8)	2.7 (0.2)	7.2 (0.3)	9.5 (0.7)	5.8 (0.7)	−22.2 (0.6)	12.5 (0.4)
231	66.0	77.5	26.9 (1.0)	23.3 (0.3)	26.6 (0.3)	25.5 (0.9)			23.9 (0.9)	22.8 (0.6)	2.7 (0.3)	6.9 (0.4)	8.5 (0.5)	6.4 (0.8)	−21.2 (0.9)	13.4 (0.3)
388	69.1	96.5	31.4 (0.3)	30.1 (0.4)	21.2 (0.1)	28.4 (0.2)			26.2 (0.3)	27.1 (0.6)	4.4 (0.6)	8.9 (0.5)	9.2 (0.2)	10.0 (0.3)	−17.5 (0.5)	14.6 (0.1)
416	69.9	85.2	32.3 (0.6)	29.8 (0.2)	29.2 (0.4)	28.5 (0.3)			25.0 (0.6)	28.6 (1.0)	4.4 (0.6)	8.6 (0.5)	11.1 (0.3)	8.3 (0.9)	−18.7 (0.4)	14.2 (0.6)
481	82.0	97.0	29.6 (0.4)	27.5 (0.5)	27.8 (0.3)	27.4 (0.4)			26.0 (0.7)	23.1 (0.6)	2.0 (0.2)	8.0 (0.3)	10 (0.1)	7.7 (0.9)	−18.0 (0.9)	12.0 (0.7)
572	65.5	89.0	29.6 (0.6)	25.2 (0.8)	26.2 (0.5)	26.2 (0.7)			24.6 (0.7)	24.1 (0.8)	6.8 (0.4)	7.2 (0.5)	6.9 (0.1)	5.6 (0.7)	−20.0 (0.9)	11.3 (0.6)
723	69.0	92.3	31.0 (0.2)	30.4 (0.5)	29.1 (0.1)	27.6 (0.3)			26.0 (0.2)	24.7 (0.4)	3.4 (0.2)	9.0 (0.1)	9.7 (0.1)	7.1 (0.2)	−19.8 (0.7)	13.6 (0.1)
1054	64.5	105.2	34.3 (0.9)	29.1 (0.2)	31.2 (0.2)	30.7 (0.7)			26.1 (0.3)	25.6 (0.4)	8.4 (0.2)	8.7 (0.8)	7.9 (0.6)	9.5 (0.6)	−17.4 (1.2)	15.1 (0.4)

Each time point is represented by an individual sample (n = 1). Curved fork length (CFL) was measured at time of capture (initial) and death. Standard deviations are calculated as the propagated error of sample injections, reflecting analytical uncertainty, and are given in parentheses. All values are shown in ‰. Essential amino acids are indicated by †.

The δ^15^N values of trophic amino acids had greater variation among components (squid, sardine, and gelatin) of the captive feed (greatest σ = 6.0‰ in Pro) than in source amino acids (greatest σ = 4.5‰ in Ser; Table 2). The smallest variation was found in the source amino acid lysine (σ = 0.3‰). The temporal variation in individual feed component amino acid isotopic compositions was mostly small (average range: trophic  = 1.1‰, source, excluding Thr  = 1.5‰); the largest variation was found in Thr values of sardine (3.62‰). Although analysis of prey items from two time points may not encompass the temporal variability within the diet, it has been shown that the δ^15^N value of source amino acids such as phenylalanine correlates significantly with the δ^15^N values of bulk tissue [8]. Data from Madigan et al. [21] did not show significant temporal changes in bulk tissue of prey items sampled at 13 time points during the diet study. Furthermore, changes in trophic amino acids are primarily driven by changes in trophic position. Despite documented changes in prey type abundances, estimated trophic positions from stomach content analyses of squid and sardine have not shown significant annual changes [33], [34], and therefore do not indicate the likelihood of a significant temporal change in the isotopic composition of trophic amino acids. The mass-weighted mean of the feed was considerably enriched in ^15^N compared to initial PBFT (day 11) in eight of the twelve amino acids, as much as 12.9‰ in Thr. Aspartic acid in feed was depleted in ^15^N relative to initial PBFT but there was no detectable difference in the ^15^N contents of Phe, Glu and Tyr (Table 2). The lack of difference in δ^15^N values between initial PBFT white muscle and the mass-weighted mean of the feed meant that turnover could be modeled in only four trophic amino acids (Ala, Leu, Pro, and Val) and four source amino acids (Gly, Lys, Ser, and Thr). Glutamic acid was also included despite this lack of difference in δ^15^N values due to its significant metabolic isotopic fractionation.

**Table 2:** Average δ^15^N values of amino acids in individual parts of the PBFT captive diet.

	Trophic Amino Acids											Source Amino Acids									
	Ala (SD)	Asp (SD)	Glu (SD)		Leu (SD)†			Pro (SD)		Val (SD)†		Gly (SD)	Lys (SD)†	Phe (SD)	Ser (SD)		Thr (SD)†		Tyr (SD)		
Squid (n = 2)	29.7 (0.4)	19.5 (1.9)		24.3 (0.3)			24.6 (0.3)		24.0 (1.4)		23.2 (0.9)	4.0 (0.1)	8.8 (0.4)	5.8 (0.2)	15.0 (1.4)		−11.3 (0.3)		14.0 (1.0)		
Sardine (n = 2)	25.7 (1.6)	17.6 (0.7)		22.9 (0.1)			23.2 (1.3)		19.7 (0.4)		24.1 (2.0)	6.9 (2.1)	8.6 (0.1)	8.1 (2.9)	8.5 (0.6)		−5.3 (3.3)		10.1 (3.0)		
Gelatin (n = 2)	18.3 (0.5)	15.2 (0.6)		18.5 (0.3)			18.5 (0.4)		12.1 (0.3)		17.4 (0.7)	5.7 (0.7)	8.2 (0.1)	7.4 (0.8)	6.4 (0.1)		−5.2 (0.7)		12.8 (0.6)		
Standard Deviation (σ)	5.8	2.2		3.0		3.2		6.0		3.6		1.5	0.3	1.2		4.5		3.5		2.0	
Mass Balance	27.4 (1.7)	18.6 (2.1)		23.3 (0.4)		23.6 (1.4)		21.6 (1.5)		22.9 (2.3)		5.1 (2.3)	8.7 (0.4)	6.7 (1.0)		12.2 (1.6)		−8.9 (3.4)		12.7 (3.2)	
Feed Enrichment	3.2 (1.9)	−4.6 (2.1)		−0.6 (0.6)		2.4 (1.5)		4.8 (1.7)		3.6 (2.3)		8.2 (2.4)	4.7 (0.5)	−1.7 (1.0)		11.8 (1.6)		12.9 (3.3)		1.4 (3.3)	

Weighted mean of the diet was calculated from the weight percent contribution of the components: 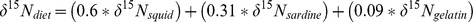. Feed enrichment reflects the change in nitrogen isotopic composition of amino acids between feed and 11 d captive PBFT. Standard deviations (SD) for each amino acid are given in parentheses, calculated from the error between diet samples and experimental error. The standard deviation of all diet components is given as σ. All values are shown in ‰. Essential amino acids are indicated by †.

### Time- and growth-based Turnover

**Figure 1:**
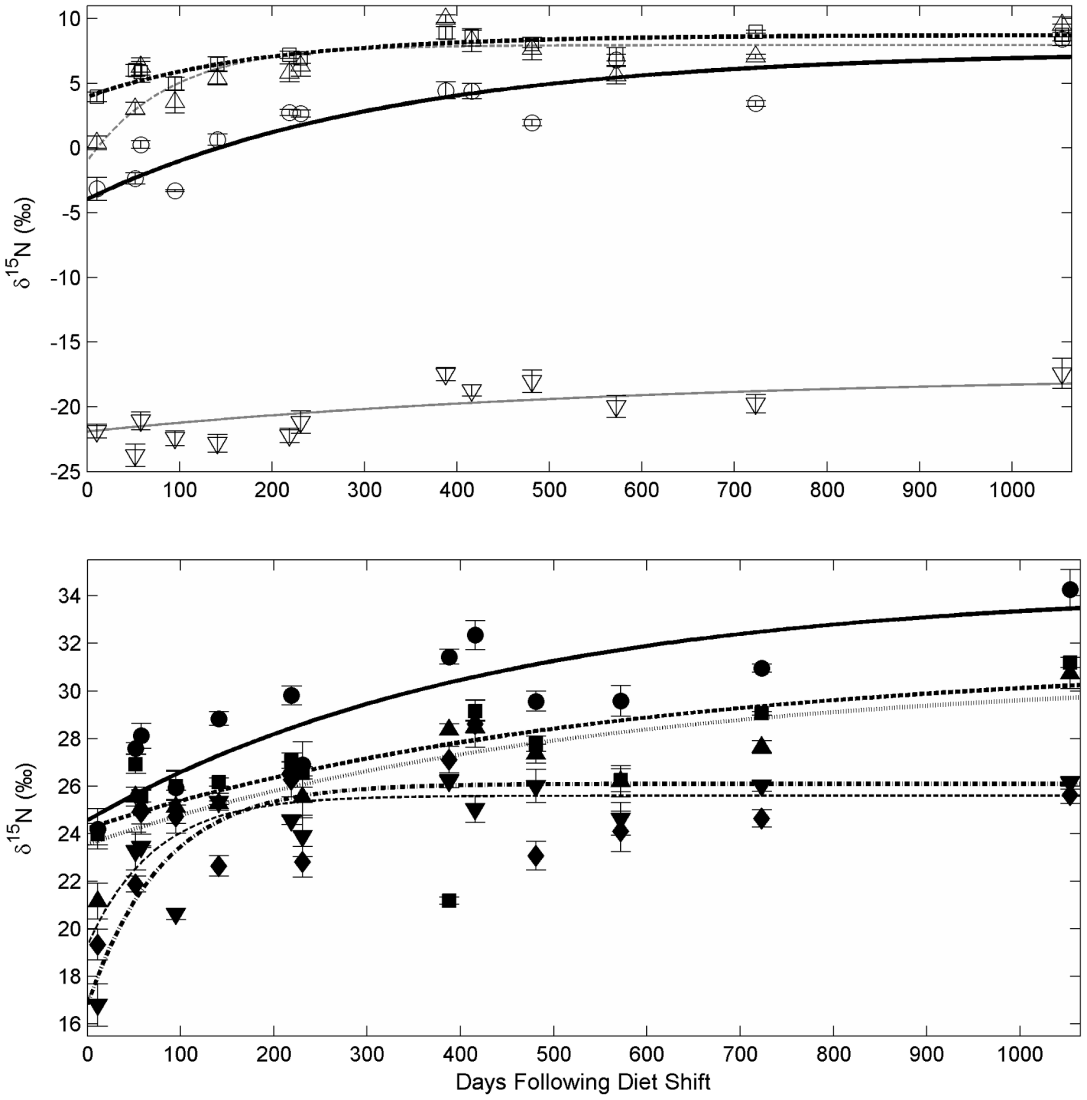
Change in amino acid δ^15^N values in white muscle in captive Pacific bluefin tuna (*Thunnus orientalis*). Isotopic change over time following a diet shift in A) four source amino acids - glycine (open circle), lysine (open square), serine (open triangle), threonine (open inverted triangle), and B) five trophic amino acids - alanine (filled circle), glutamic acid (filled square), leucine (filled triangle), proline (filled inverted triangle), and valine (unfilled diamond) in Pacific bluefin tuna white muscle tissue. Lines represent best fit lines for an exponential fit model.

Turnover was evident within the first 800 days in trophic and source amino acids, after which asymptotic values (steady-state) were approached (Fig. 1). Despite differences in initial values, source amino acid δ^15^N values converged at steady-state to within 1.1‰ of each other, with the exception of Thr which was depleted in ^15^N relative to the other source amino acids. Other than Ala, trophic amino acids which had similar starting δ^15^N values (initial PBFT δ^15^N) approached similar asymptotic values. Overall, exponential models described amino acid incorporation reasonably well, with only Val returning a relatively poor fit (r^2^ = 0.44, Table 3). Turnover rates varied over an order of magnitude within both trophic and source amino acids, with calculated half-lives ranging from less than two months (Pro, Val) to over one year (Glu, Thr). The fastest turnover times were found in two trophic amino acids (t_0.5_Val = 50 d and t_0.5_Pro = 55 d), while the slowest turnover was in Thr (t_0.5_ = 425 d), considered a source amino acid [4]. No consistent trends were obvious between groupings of trophic versus source or essential versus nonessential amino acids.

**Table 3:** Time-based ^15^N amino acid incorporation rate, half-life (t_0.5_) and steady state (t_0.95_) estimates, in days, with 95% confidence intervals calculated from an exponential fit model for Pacific bluefin tuna (*Thunnus orientalis*).

AA		λ (95% CI)	r^2^	*t_0.5_* (95% CI)	*t_0.95_* (95% CI)
**Trophic**	Ala	0.0023 (0.0011, 0.0036)	0.63	297 (195, 622)	1283 (843, 2687)
	Glu	0.0018 (0.0001, 0.0035)	0.60	387 (199, 6533)	1671 (861, 28235)
	Leu†	0.0019 (0.0008, 0.0030)	0.67	369 (234, 863)	1593 (1013, 3731)
	Pro	0.0126 (0.0038, 0.0214)	0.70	55 (32, 182)	238 (140, 786)
	Val†	0.0140 (0.0018, 0.0263)	0.44	50 (26, 395)	214 (114, 1709)
**Source**	Gly	0.0023 (0.0012, 0.0033)	0.78	307 (212, 558)	1326 (915, 2410)
	Lys†	0.0053 (0.0009, 0.0096)	0.81	131 (72, 734)	567 (311, 3171)
	Ser	0.0048 (0.0016, 0.0081)	0.57	143 (86, 433)	619 (371, 1874)
	Thr†	0.0113 (0.0003, 0.0223)	0.56	425 (248, 1463)	1836 (1073, 6324)

Essential amino acids are indicated by †.

Assuming steady state after 95% turnover was achieved in isotopic composition, the majority of amino acids, both trophic and source, required at least 3.5 years to reach isotopic steady-state with the diet. All amino acids except Thr reached steady-state within estimated confidence intervals (CIs) by the final time point analyzed here (1054 d).

**Figure 2:**
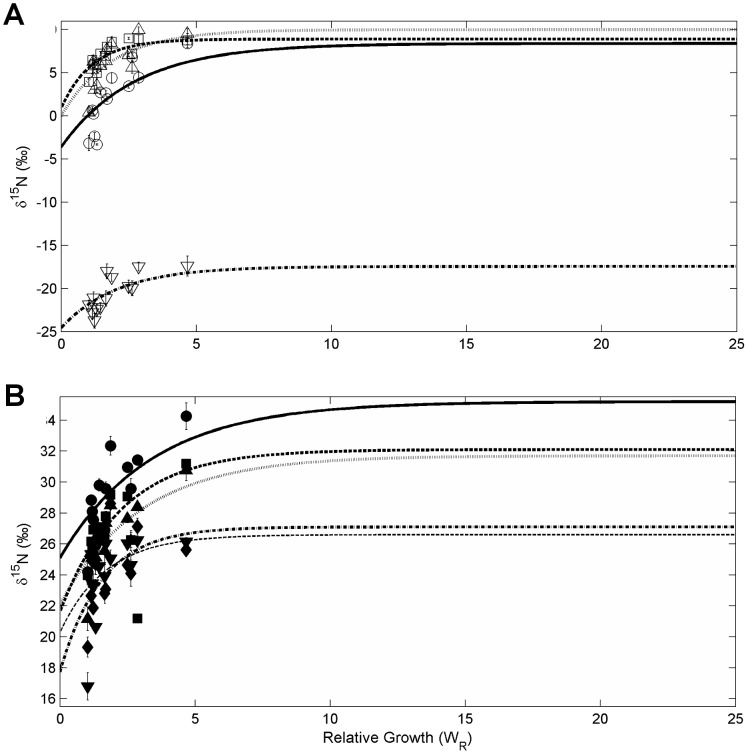
Growth based changes in amino acid isotopic composition in captive Pacific bluefin tuna (*Thunnus orientalis*). Incorporation of ^15^N into amino acids of white muscle tissue as a function of relative growth (W_R_). A) Four source amino acids, glycine (unfilled circle), lysine (unfilled square), serine (unfilled triangle), threonine (unfilled inverted triangle), and B) five trophic amino acids - alanine (filled circle), glutamic acid (filled square), leucine (filled triangle), proline (filled inverted triangle), and valine (unfilled diamond) are given. Lines represent best fit lines for an exponential fit model.

Growth-based turnover for nitrogen in amino acids followed similar patterns to time-based models. Trophic amino acids pro and val and source amino acids Lys and Ser had the shortest growth-based half-lives (G_0.5_), although Pro and Val were not well described by the exponential fit model (Table 4). Generally, trophic amino acids took longer to approach isotopic steady state with the diet than did source amino acids (Fig. 2) and given growth alone, none did within the time of the experiment. Unlike time-based models, Ala, a trophic amino acid, required the most growth (120%), while Glu and Thr needed only 60% and 50% mass increase, respectively, for 50% turnover.

**Table 4:** Amino acid ^15^N incorporation rates and growth-based half-life (G_0.5_) estimates for exponential fit models of relative growth (W_R_) with 95% confidence intervals in Pacific bluefin tuna (*Thunnus orientalis*).

AA		λ (95% CI)	r^2^	G*_0.5_* (95% CI)
**Trophic**	Ala	0.31 (0.19, 0.43)	0.55	3.2 (4.6, 2.6)
	Glu	0.44 (0.12, 0.75)	0.62	2.6 (6.6, 1.9)
	Leu†	0.34 (0.24, 0.43)	0.60	3.1 (3.9, 2.6)
	Pro	0.64 (0.37, 0.92)	0.36	2.1 (2.9, 1.8)
	Val†	0.67 (0.25, 1.08)	0.21	2.0 (3.7, 1.6)
**Source**	Gly	0.38 (0.24, 0.52)	0.62	2.8 (2.3, 3.9)
	Lys†	0.71 (0.48, 0.94)	0.57	2.0 (1.7, 2.4)
	Ser	0.54 (0.37, 0.71)	0.50	2.3 (2.0, 2.9)
	Thr†	0.47 (0.30, 0.63)	0.54	2.5 (2.1, 3.3)

Essential amino acids are indicated by †.

### Reaction-Progress Variable

**Figure 3:**
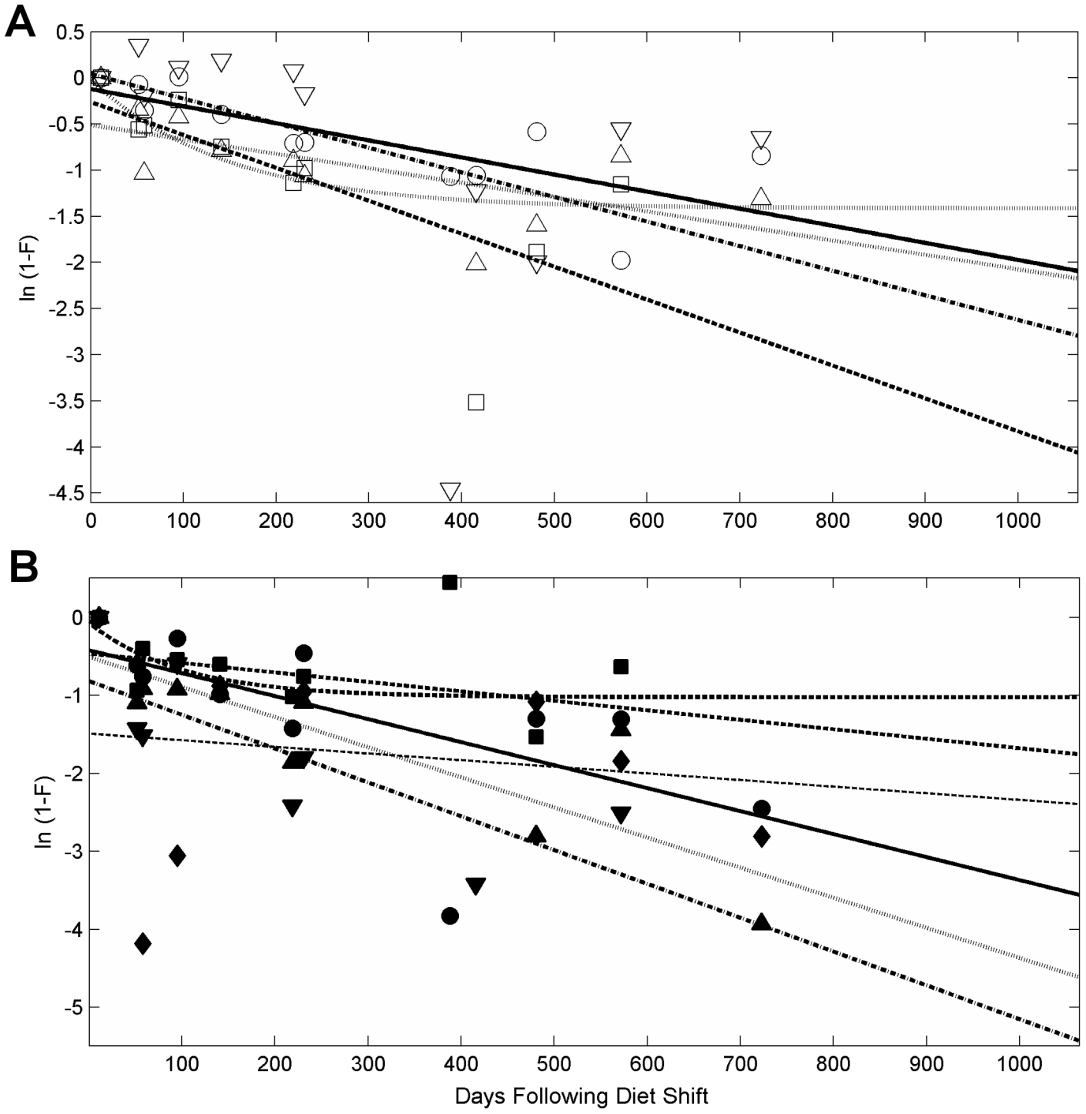
Reaction-progress variables for isotopic changes of amino acids in captive Pacific bluefin tuna (*Thunnus orientalis*). Changes in isotopic composition over time of A) four source amino acids, glycine (unfilled circle), lysine (unfilled square), serine (unfilled triangle), threonine (unfilled inverted triangle), and B) five trophic amino acids - alanine (filled circle), glutamic acid (filled square), leucine (filled triangle), proline (filled inverted triangle), and valine (unfilled diamond). Lines represent best fit for a linear fit model, with additional exponential fit models for proline and serine.

**Figure 4:**
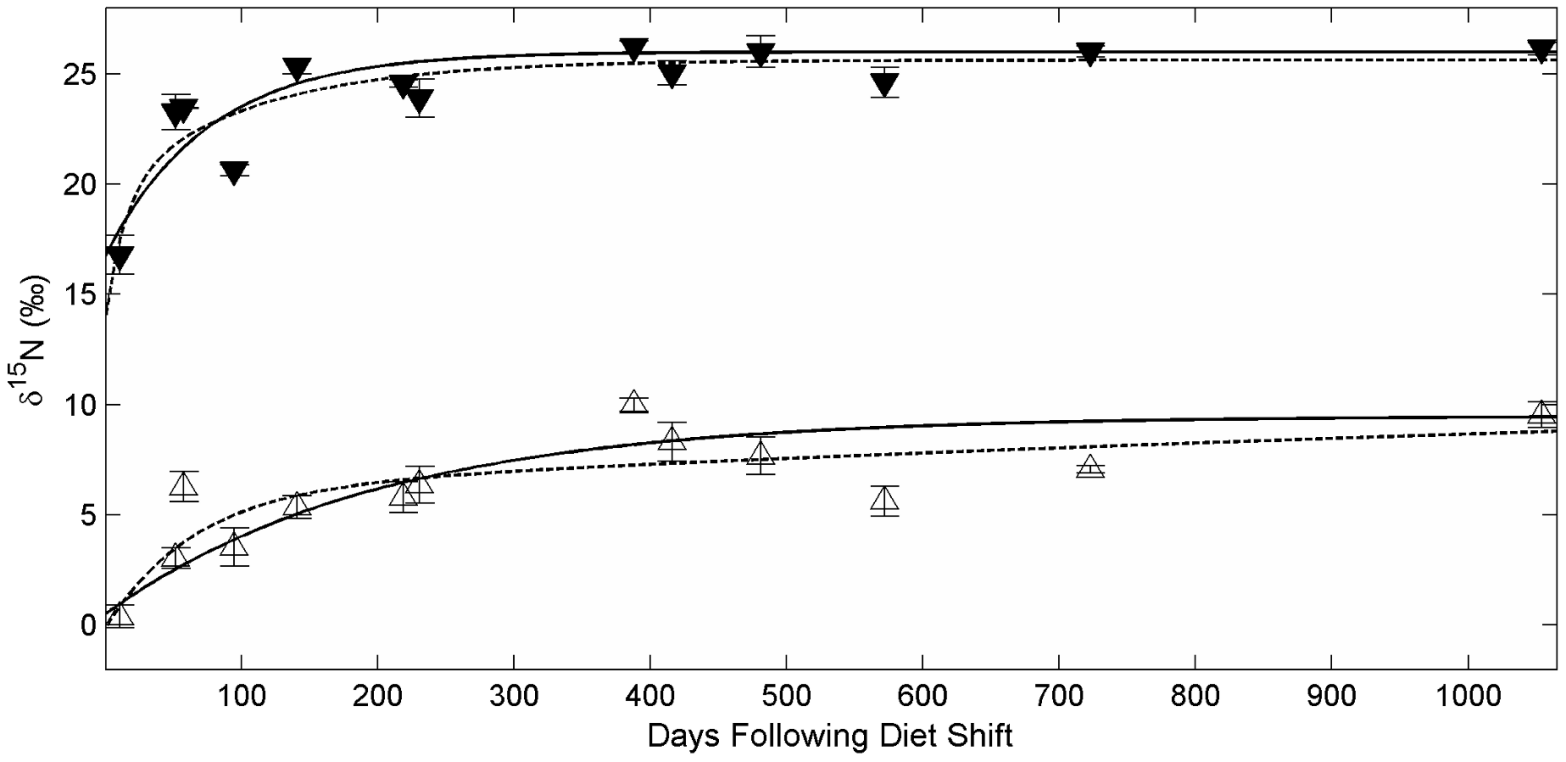
Multi-compartment exponential fit model of amino acid isotopic composition of Pacific bluefin tuna (*Thunnus orientalis*). Changes of δ^15^N values of proline (filled inverted triangle), a trophic amino acid, and serine (unfilled triangle), a source amino acid in white muscle tissue of Pacific bluefin tuna as a function of time. Solid lines represent the single compartment exponential fit model and dashed lines include a second compartment within the model as described by Cerling et al. (2006).

A log transformation of the data to calculate the reaction progress variable resulted in the majority of amino acids being best described by a linear function of ln(1-F) decreasing with increasing time, and therefore a single compartment model (Fig. 3). The goodness of fit of log transformed results versus time for pro and ser were improved using an exponential fit (r^2^ = 0.72 and 0.59, respectively), and were therefore improved by a multi-compartment model fitted to the untransformed data (Fig. 4). Val was not described well by either fit and the intercept of the fit was much less than zero. Reaction-progress derived half-lives of amino acids were different than those derived from time-based exponential model fits (Table 5), generally resulting in longer turnover times for all amino acids. Compared to the exponential model, the linear model yielded a much shorter turnover times for Leu and Thr (t_0.5_ = 180 d and 260 d, respectively) and longer turnover times in Glu and Lys (t_0.5_ = 571 d and 194 d, respectively). Estimated half-lives of Pro and Ser were approximately 3 times longer.

**Table 5:** Parameters with 95% confidence intervals for a linear fit model of the reaction-progress variable for individual amino acids.

AA		m	b	r^2^	t_0.5_
**Trophic**	Ala	−0.002 (−0.004, 0.001)	−0.3 (−0.8, 0.1)	0.72	287
	Glu	−0.001 (−0.003, 0.000)	−0.5 (−0.9, 0.0)	0.31	571
	Leu†	−0.004 (0.006, −0.002)	−0.5 (−1.2, 0.2)	0.73	180
	Pro	−0.004 (−0.008, −0.001)	−0.8 (−1.8, 0.1)	0.62	160
	Val†	−0.001 (−0.005, 0.004)	−1.5 (−3.2, 0.2)	0.03	815
**Source**	Gly	−0.002 (−0.003, −0.001)	−0.1 (−0.5, 0.3)	0.57	384
	Lys†	−0.004 (−0.007, −0.001)	−0.3 (−1.1, 0.6)	0.49	194
	Ser	−0.002 (−0.003, 0.000)	−0.5 (−1.0, 0.0)	0.43	443
	Thr†	−0.003 (−0.006, 0.001)	0.5 (0.3, 0.6)	0.20	260

Essential amino acids are indicated by †.

### Amino Acid Specific TDF

TDF values for nine amino acids were calculated using *c* in eqn. 2 for fish that had reached isotopic steady-state with their diet (Table 6). Because phenylalanine did not change between diets, as evidenced by the lack of isotopic change between initial PBFT and diet, it was assumed to be in steady-state with both the natural and experimental diet and therefore included in the analysis by using the average values of all tuna analyzed. Trophic amino acid TDFs were higher than source TDFs, ranging from 2.3±0.3 (Val) to 7.8±0.2 ‰ (Glu) versus −8.5±0.2 (Thr) to 3.4±0.2‰ (Gly), respectively. All amino acids other than Ser, which was depleted in ^15^N compared to the diet, were near or within the error of TDFs calculated for individual amino acids in previous publications [4], [7].

**Table 6:** Data-derived steady-state nitrogen isotopic composition and TDF values of individual amino acids in Pacific bluefin tuna (*Thunnus orientalis*).

AA		Sample size	Steady State (SD)	TDF (SD)	TDF McClelland and Montoya 2002 (SD)	TDF Chikaraishi et al. 2009 (SD)
**Trophic**	Ala	1	34.3 (0.9)	6.8 (0.9)	5.0 (0.8)	6.1 (2.1)
	Glu	1	31.2 (0.2)	7.8 (0.2)	6.7 (0.6)	8.0 (1.2)
	Leu†	1	30.7 (0.7)	7.1 (0.7)	3.4 (0.9)	4.8 (2.0)
	Pro	6	25.7 (0.2)	4.1 (0.8)	4.0 (0.5)	6.1 (1.6)
	Val†	8	25.3 (0.2)	2.3 (0.3)	3.7 (1.6)	5.0 (1.7)
**Source**	Gly	1	8.4 (0.2)	3.4 (0.2)	0.9 (1.0)	3.7 (3.9)
	Lys†	3	8.3 (0.3)	−0.3 (0.4)	1.6 (1.3)	n/a
	Ser	2	8.3 (0.3)	−4.2 (0.3)	0.8 (0.8)	3.6 (3.0)
	Phe†	13	8.7 (0.2)	1.5 (0.3)	0.3 (1.1)	0.4 (0.5)
	Thr†	1	−17.4 (0.0)	−8.5 (0.2)	−1.4 (0.8)	n/a

TDF were calculated using fish that had achieved 95% turnover within confidence intervals determined through time-based models. TDF values for a wide range of species reported by McClelland and Montoya (2002) and Chikaraishi et al. (2009) are shown. Essential amino acids are indicated by †.

### Comparison to wild data

**Figure 5:**
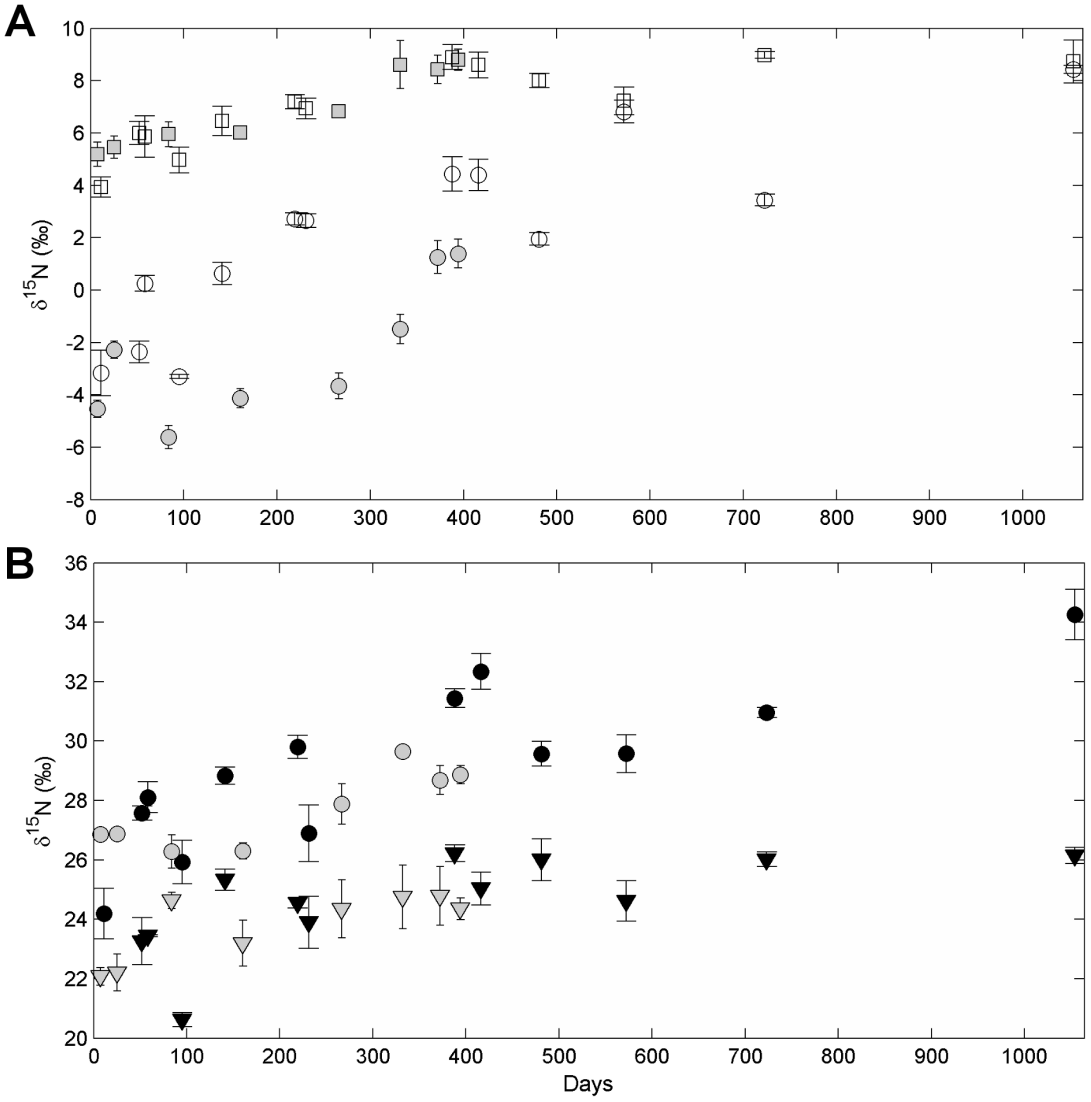
Comparison of amino acid isotopic composition in wild and captive Pacific bluefin tuna (*Thunnus orientalis*). Change in δ^15^N values of amino acids of white muscle tissue with time following a natural diet shift. A) Two source amino acids, glycine (unfilled circle), lysine (unfilled square), and B) two trophic amino acids - alanine (filled circle), proline (filled inverted triangle) are given. Wild samples are shown as grey shaded symbols.

Isotopic turnover rates of six of the nine amino acids were similar between wild Pacific bluefin tuna and captive fish. Source amino acids, Gly and Ser, and the trophic amino acid Ala, were all depleted in ^15^N in wild PBFT. Examples are shown in Fig. 5. Data were not available for wild PBFT of comparable size to the largest PBFT in the captive study.

## Discussion

Differences between isotopic compositions of nine individual amino acids in white muscle tissue of early immigrant PBFT and their captive diet were high enough to result in an observable shift in ^15^N contents over time, which yielded information about the incorporation rates and ^15^N discrimination in amino acids. The range of time for amino acid 95% turnover (Val: 214 d to Thr: 1836 d) shown in this study illustrates the importance of diet studies spanning multiple years, potentially longer than those required for bulk tissue to reach complete turnover.

### Assumptions of 1^st^ order kinetics and the single compartment model

The use of the reaction progress variable (RPV) has been advocated as a diagnostic tool to gauge the appropriateness of one- or multi-compartment models of incorporation [13], [30], [35]. Results of several recent studies of bulk muscle tissue have shown that isotopic turnover of both carbon and nitrogen can be most often described by a single compartment model, and rarely requires more than two compartments [15], [21], [30]. The potential for a multi-compartment model for the incorporation of amino acids exists in that metabolism and synthesis of amino acids can be governed by a number of processes, leading potentially to multiple pathways with different incorporation rates. Results of this study indicate that perhaps only one or two pathways are dominant for each amino acid. The linear fit of the individual AA δ^15^N values to the RPV indicates a single compartment model for most amino acids (Fig. 3), as was also seen from results of bulk tissue [21]. Two amino acids, Ser and Pro, were better described with a two-compartment model (r^2^ = 0.81 and 0.71, respectively), suggesting influence from multiple metabolic pathways, although these data also fit relatively well to a single compartment model (r^2^ = 0.73 and 0.61, respectively) and the paucity of data do not justify using a more complex model. Therefore, incorporation of ^15^N in the amino acids in white muscle protein over time and growth were described by an exponential fit, single compartment model following first order kinetics.

### Amino acid isotopic incorporation and turnover

The rate of ^15^N incorporation as a function of time is influenced by both metabolism and growth (time-based model). However, assuming that addition of mass is directly proportional to the addition of muscle tissue, examining the rate of ^15^N incorporation as a function of relative change in mass should be influenced mainly by the rate of organism growth (growth-based model). A subsequent comparison of turnover calculated from these two models can allow separation of the main factors controlling isotopic turnover. Here we also assume that the increase in concentration of each individual amino acid is similar to one another and proportional to muscle growth in order for quantitative conclusions about the effect of growth on isotopic incorporation to be drawn. Since changes in concentrations of specific amino acids are unknown from this study, time based models are the primary basis for interpreting amino acid isotopic turnover. Comparisons to growth models provide insight but can only allow estimates of the relative contributions of growth and metabolism.

The rate of ^15^N incorporation into amino acids in white muscle protein may be affected by a number of processes – whether or not an organism can synthesize the AA *de novo* (essential or nonessential), the kinetic isotope effects associated with transport of the AA (molecular weight and structure of the amino acid), and the biochemical reactions controlling metabolic breakdown, which we assume affects trophic and source AA differently (Chikaraishi et al. 2007, McCarthy et al. 2013). The grouping of essential (EAA) and non-essential (nEAA) amino acids has been previously shown to have no relationship with δ^15^N values in consumers [4], [6], [36]. Similarly, the results of this study found no relationship between amino acid half-life and the EAA – nEAA grouping in either time or growth based models.

Smaller AAs are expected to break down and form more easily and rapidly, resulting in reduced fractionation and faster turnover rates. However, neither structural complexity nor molecular weight appeared to correlate with amino acid turnover rates. Glycine, the smallest and structurally least complex amino acid, had an intermediate turnover time (t_0.5_ = 307 d) compared to others, whereas proline, a rigid cyclic amino acid, was among the quickest (t_0.5_ = 55 d). The rapid turnover of Pro in both time and growth models, and its better fit to a two compartment model, reflect its dual uptake pathways (Fig. 4); it is assimilated both through passive diffusion and active transport, via oxidative metabolism, generating ATP [37]. The intermediate turnover time of glycine may reflect a synthetic pathway from carbohydrate or lipid, rather than protein, a pathway that has been suggested in trout, evidenced by a subsequent enrichment of glycine ^13^C when diets contained carbohydrates or lipids enriched in ^13^C [38]. An absence of dietary protein derived Gly directly assimilating into consumer protein in PBFT could likely lead to the lengthened incorporation rates seen in glycine despite its simple structure.

Tissues with high rates of metabolism, such as liver, display rapid isotopic turnover [21], [39], [40], and isotopic fractionation due to metabolic breakdown has been proposed as the mechanism separating trophic from source amino acids [4], [6], [7]. It was expected that the more metabolically active trophic amino acids would have more rapid isotopic incorporation compared to source amino acids. Although the fastest turnover times occur in the trophic amino acids (Pro,Val) and the slowest turnover was observed in a source amino acid (Thr), the half-lives of amino acids ranged considerably, resulting in no correlations with the trophic or source grouping (Table 3). In general, the incorporation of ^15^N into amino acids as a function of growth shifted incorporation rates such that both source and trophic amino acids had similar growth-based half-lives (Table 4). This impact was strongest on trophic amino acids, reducing turnover times by as much as a seven-fold decrease (Pro, Val). The importance of metabolism on the turnover of trophic amino acids is evident in more rapid incorporation rates seen in time-based models. This was expected given previous evidence of metabolic breakdown being a driving force in the isotopic fractionation seen in trophic amino acids [6].

Special emphasis has been given to glutamic acid in previous isotopic studies, as it has a considerable ^15^N enrichment with each increase in trophic level [6], and is a major amino acid utilized as a catalytic carrier of nitrogen to the liver [37], [41]. In studies of the absorption rates of amino acids in PBFT intestines, Glu had consistently high rates and percentages of assimilation [37], [41] leading to the expectation that isotopic incorporation would be rapid. Results for the turnover of Glu yielded intermediate turnover times in both time and growth models (Tables 3 & 4). Upon transport to the liver, the nitrogen from Glu can be utilized in the formation of any number of amino acids, leading to a grouping of AAs with similar nitrogen isotopic compositions which appear to be coupled to Glu [36]. In this study, Ala, Glu, and Leu had steady state δ^15^N values within 3.6‰ of each other, while Val and Pro were depleted in ^15^N by 5‰ relative to the other three (Table 6). Similarly, incorporation rates of Ala, Glu, and Leu were comparable to one another (λ = 0.0023, 0.0018, 0.0019, respectively) but considerably different than those of Val and Pro (λ = 0.0126, 0.0140, respectively). These results could indicate a linkage of Ala and Leu with Glu, while different biochemical processes are responsible for the synthesis of Val and Pro.

Comparison to natural samples can provide confidence in the results of captive diet studies. In this study, only a qualitative assessment was appropriate, as wild PBFT in steady state with their new diet were not available. Several amino acids did show similar values and turnover rates between wild and captive samples (Fig. 5), supporting the half-lives calculated here. While the results from most AAs show that this study reasonably estimated turnover for some amino acids, it is evident that further understanding of the PBFT diet is needed. Three amino acids, Ala, Gly, and Ser, which share a common metabolite, pyruvate, were depleted in ^15^N in wild samples, suggesting that those amino acids were obtained from a food source that was not represented in the captive diet. Given that these three AAs are nonessential, it is possible that they were synthesized *de novo* in the wild fish, leading to lower δ^15^N values in recent immigrants.

### Trophic Discrimination Factors (Δ)

The use of AA-CSIA in trophic studies to calculate trophic position requires specific knowledge of the fractionation of ^15^N in each amino acid between food and consumer, known as the trophic discrimination factor (TDF), or Δ_AA_. Trophic position is estimated using the difference between Δ_AA_ of a single or group of trophic and source amino acids, referred to in the literature as a trophic enrichment factor, or TEF [5], [7], [8], [42], [43]. It is becoming increasingly more important to accurately calculate Δs for multiple amino acids, as reliance upon only a single representative trophic and source amino acid can be susceptible to error [11], [44].

Trophic discrimination factors (Δs) have been reported for multiple amino acids in a number of lower trophic position organisms [4], [6], [7]. In this study, Δ_AA_s for PBFT resemble those previously published, except Δ_Ser_ (-4.2±0.3‰), which has been shown previously to have a significant range of values across trophic levels [7]. Δ_Thr_ was also considerably more negative than recently reported (Table 6), however, earlier experiments have found a 6 ‰ depletion in ^15^N per trophic step [45], more closely resembling these data. The considerable depletion of Thr has been suggested to be the result of an inverse isotope effect created by threonine deaminase [45], [46]. In most species, serine deaminase is identical to threonine deaminase, and may have a similar effect during the catabolism of serine. Our results confirm that trophic amino acids show considerable ^15^N enrichment relative to those same amino acids in their diet and also are in agreement with results of previous studies that show minimal ^15^N enrichment in source amino acids relative to those in their diet. It has been suggested that Phe represents the most consistent source amino acid with the smallest isotopic fractionation during trophic transfer [7], [36]. Here we found Δ_Phe_ to be 1.5±0.3 ‰, which was larger than previous accounts, but still smaller than that seen in trophic amino acids. Additionally, Lys actually had a near zero isotopic change, indicating that it may also serve as a representative source amino acid.

Increasingly, a TEF of 7.6‰ between Glu and Phe has been used in trophic calculations in isotopic studies, a value which has been applied to a wide range of trophic levels studied [7], [8], [31], [42], [43]. The original calculation of this value was made from diet studies of three trophic levels [7], and has been both confirmed [31] and questioned [42], [43] in samples collected from higher trophic level, wild organisms. In this study, the TEF_Glu-Phe_ is less than previously published (6.3±0.4‰) as is the TEF calculated from a weighted mean of trophic and source amino acids (5.0±1.5‰). These findings support recent publications which indicate that a TEF of 7.6‰ consistently underestimates trophic level in trophic calculations and propose reduced TEF values for higher-TL organisms [42], [43].

Given that bluefin tuna have a higher trophic position than the study organisms previously used to calculate TEF_Glu-Phe_, our results may imply that enrichment between trophic and source amino acids decreases with increasing trophic position. If this is true, it may be the result of the increased protein content of diets of carnivores, which has been shown to correspond to differential isotopic fractionation between food and consumer compared to that of herbivores [40], [47]. Additionally, altered metabolic activity levels or pathways may be in part driving forces for differential fractionation between trophic and source amino acids. From the results of incorporation rates, this study demonstrated that metabolism can have different effects on the turnover of trophic or source amino acids (Tables 3 & 4), which could potentially lead to differential isotope fractionation. It is possible that the effects of captivity result in variability in isotopic fractionation, although previous studies have shown similar values derived from both captive and wild caught samples [6], [7], [31]. The wild PBFT from this study had not reached steady-state with their new diet, therefore a comparison of TEF could not be made. However, the reduced TEF values calculated here are similar to those back calculated from other wild samples [42], suggesting that captivity was not a likely cause of error.

### Implications for trophic studies

**Figure 6:**
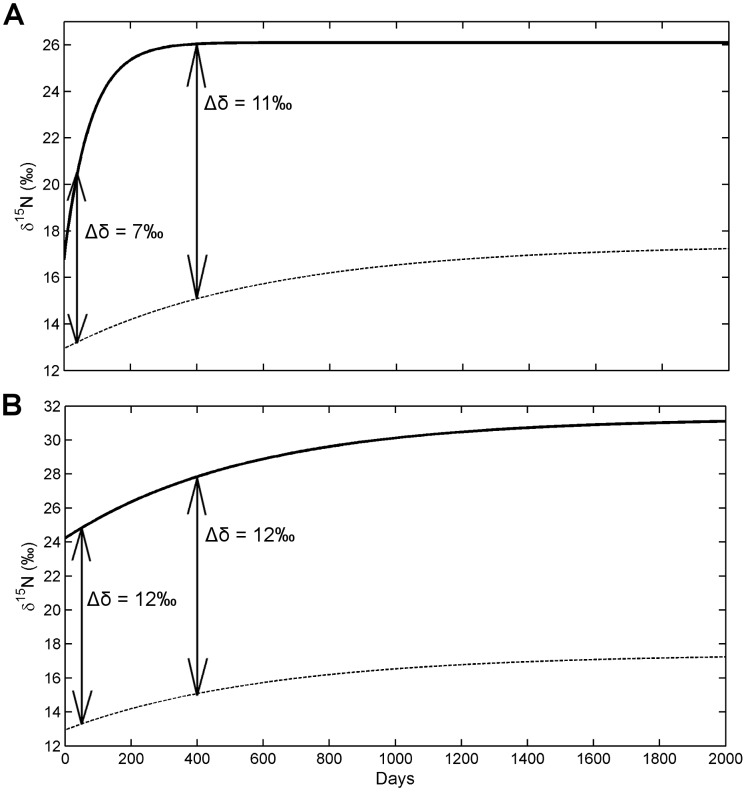
Schematic of fractionation between amino acids following a diet shift with variable incorporation rates. Difference in δ^15^N values of two combinations of source and trophic amino acids. A) Isotopic change between source (dashed line) and trophic (solid line) amino acids with dissimilar turnover rates at two time points compared to B) those of source (dashed line) and trophic (solid line) amino acids turning over at similar speeds.

Results given here are the first for amino acid incorporation rates of a long-term diet study. The impact on current and future trophic studies is multi-fold and potentially far reaching. While the disparities in turnover times between source and trophic amino acids allow for distinction of immigrants from residents, they could prove problematic in calculating trophic position using isotopic composition of migrating PBFT or recent immigrants. Using amino acids that have significantly different turnover rates, such as the combination of Pro and Thr, the AA with longest and shortest turnover times, respectively, trophic level calculations will vary significantly depending on how close an organism is to reaching steady-state with its diet. In this case, trophic level (TL) could vary as much as one trophic position across the duration of the study. This is illustrated in Fig 6a and shows that the difference in each amino acids turnover rate is reflected in variable estimates of trophic position within the first 480 days of captivity, or following a change in the ^15^N contents of their diet, until the half-lives are reached for both amino acids. In contrast, the trophic-source combination of Glu and Thr, which have similar half-lives, yields significantly less variability in TL estimates, only 0.3 trophic positions (Fig. 6b). From the results of this study, the trophic-source combinations of Ala and Gly or Glu and Thr will provide the most accurate estimate of trophic position regardless of immigration status. On the other hand, examining the difference in δ^15^N values between Pro and either Gly or Thr, immigrant PBFT can potentially be distinguished by deviations from published or expected trophic-source enrichment values. Determining and utilizing the amino acids which have comparable turnover rates, or using only those which are in steady-state with the diet, will yield more reliable trophic position estimates and subsequent understanding of trophic ecology of an organism.

The variable incorporation rates of amino acids in white muscle tissue of PBFT may provide the framework to create an isotopic clock, much as has been done previously with isotopic compositions of multiple tissues [19], [48], [49]. The benefit of the retrospective nature of isotopic clocks enhances electronic tagging by creating a complete pattern of movements of an organism. In this study, the large range in confidence intervals surrounding incorporation parameters due to variation in individual tuna samples precluded the development of a reliable isotopic clock. However, this limitation may be overcome as more organisms are studied.

Long-term observations of isotopic incorporation following a diet change are required to gain understanding of and apply stable isotope analysis tools to ecosystem studies of PBFT and, likely, other large pelagic fish [21], [50]. This study consisted of a single species fed under controlled conditions with comparisons to wild caught samples, and extension of the results to other taxa may be limited. Natural variations seen in the physiology and diets within and among populations can contribute to variability in turnover rates. Still, our results indicate that the current simplistic view of the biochemistry governing isotopic fractionation of trophic and source amino acids may be incomplete, as ^15^N incorporation rates do not follow a similarly simple pattern. Additionally, inaccuracies in the estimation of TEF in high order marine organisms further point to possible gaps in our knowledge of the relationship between amino acid metabolism and isotopic fractionation. While it is very clear that more information is required, results from long-term studies such as those presented here are beginning to provide invaluable insights into movement patterns and trophic ecology of marine organisms.
